# A survey of the use of complementary medicine by a self-selected community group of Australian women with polycystic ovary syndrome

**DOI:** 10.1186/1472-6882-14-472

**Published:** 2014-12-06

**Authors:** Susan Arentz, Caroline Anne Smith, Jason Anthony Abbott, Alan Bensoussan

**Affiliations:** National Institute of Complementary Medicine (NICM), University of Western Sydney, Sydney, Australia; School of Women’s and Children’s Health, University of New South Wales, Randwick, Australia

## Abstract

**Background:**

Polycystic ovary syndrome (PCOS) is a complex reproductive endocrinopathy affecting up to 20% of reproductive aged women. Whilst there are effective pharmaceutical treatment options, women with PCOS have expressed a strong desire for alternatives. This study investigates the use and attitudes of women with PCOS towards complementary medicine (CM).

**Methods:**

Women as members of PCOS support groups responded to an anonymous on-line survey which examined rates and patterns of use for CM’s, areas of health for use, perceptions of effectiveness, safety and demographic features. Data collection targeted women with PCOS using two consumer support groups. The first group self-selected following direct email to members of a land based consumer support group, the Polycystic Ovary Syndrome Association of Australia (POSAA). The second sample was generated through the electronic social network Facebook, using a snowball technique. Two surveys, identical in content, were collected by cloud based Survey Monkey. Data were described and associations between the variables, ‘reasons for use’ and ‘perceptions of effectiveness’ were explored. Non-response bias was assessed using a continuum of resistance model.

**Results:**

493 women participated in the study; 91.1% response rate from the POSAA group. Over 70% reported use of complementary medicine, usually nutritional and herbal supplements and 76.6% of CM users reported consultation with a complementary practitioner. Many participants were using CM to treat PCOS however most were using it to concurrently treat a range of health conditions, describing women’s desire for more than single symptom management. Disadvantages for CM use were cited by 71% of respondents. Women using complementary medicine with specific treatment goals in mind reported greater self-perceived effectiveness, suggesting that informed use may improve women’s satisfaction with CM. Adverse reactions were reported by 12.2% of women and the need for further research into adverse reactions for CM’s was identified. Demographic and PCOS characteristics were similar to clinical populations of PCOS and non-response bias was shown as not significant.

**Conclusion:**

This study describes the prevalence of use for complementary medicine by women with PCOS as over 70% and adds to our understanding of women’s experiences with CM and their motivations for use of CM.

**Electronic supplementary material:**

The online version of this article (doi:10.1186/1472-6882-14-472) contains supplementary material, which is available to authorized users.

## Background

Polycystic ovary syndrome (PCOS) is a complex endocrine disorder affecting up to one in five reproductive aged women [1]. PCOS is a life-long condition associated with significant endocrine, reproductive and psychological morbidity and serious long term health risks [2–4]. The exact cause is unknown however it is believed to result from multiple interactions between genes, the in utero environment and lifestyle behaviors [4]. Current treatments are only moderately effective at controlling symptoms and preventing complications [2] and women with PCOS have expressed strong dissatisfaction towards pharmaceutical therapies. In a survey of women with PCOS (n = 657), 99% indicated they would prefer to use effective and safe alternative treatments to birth control pills and fertility drugs for the management of their symptoms [5].

Women’s preferences for complementary treatments coincide with the increasing prevalence for complementary medicine (CM) use. In a critical review of 14 studies, the prevalence of CM use by pregnant women was shown to range up to 87% [6]. Other studies have demonstrated similar rates of CM use by women attending family planning clinics [7] and by menopausal women [8]. The landmark Australian Women’s Health longitudinal study of 11,454 women highlighted that nearly one third of middle aged women had consulted a complementary practitioner (chiropractor, naturopath, acupuncturist or herbalist) within the previous twelve months [9].

To date there have been no investigations into the use of CM by women with PCOS. This study aimed to examine the prevalence of use, the views and patterns of CM use including women’s attitudes, views of CM effectiveness and self-reported adverse events from CM.

## Method

Study participants completed an on-line, web-based questionnaire which examined demographic features, patterns of complementary medicine (CM) use, use of CM relating to symptoms of PCOS, perceptions of effectiveness and safety, sources of information on CM and concurrent use of CM with western medicine.

Women with PCOS were recruited through two approaches; (i) convenience sampling of women belonging to the support/advocacy group Polycystic Ovary Syndrome Association of Australia (POSAA) and (ii) a snowballing technique and response to an invitation posted on the social network site Facebook with a link to the ‘University Research for PCOS’ Facebook group. Self-referrals were received from the following Facebook groups based in two metropolitan areas in Sydney and New South Wales; PCOS Cysters (121 members); PCOS TTC (333 members); and PCOS Talk (2011 members). This study was approved by the Human Research Ethics Committee at the University of Western Sydney (EC00314) reference H9341.

### The research instrument

We defined CM from an adaptation of the Cochrane Collaboration operational definition [10]. Specifically, our definition for CM was ‘alternative or natural therapies or medicines including taking dietary supplements (herbal, vitamins, minerals and/or food supplements, for example fish oils or barley greens) and or consulting traditional, alternative and complementary practitioners’. Participants were informed about specific types of CMs at the beginning of relevant questions with an extensive list of options and an open question for ‘others’ at the end. Two prevalence questions included the option ‘prescribed by a doctor’ to identify mainstream use rather than complementary medicine use. A six point Likert scale ranging from ‘daily’ to ‘as required’ enabled participants to report their frequency of use. Participants were asked their reasons for using complementary medicine and could indicate their response to fourteen symptom options. Responses were not limited to one option. Questions about signs and symptoms of PCOS and current management preceded CM use and demographic characteristics. (Additional file 1. The survey instrument.) The research tool was tested on a pilot sample of eight women including four with PCOS.

### Survey administration

The questionnaire was administered using Survey Monkey [11]. An email containing a letter of invitation and an electronic link to the questionnaire was sent to all members of POSAA in November 2011. Two reminders were sent out at six-week intervals. The second approach used to distribute the questionnaire to the community utilized a Facebook group created by the researcher (SA) in February 2012. Facebook groups are virtual communities of choice, linking people with shared interests, attributes or causes. Wall postings occurred bi-monthly during the study administration.

### Data analyses

Analysis was undertaken using the statistical package, SPSS version 21.0 [12]. Categorical responses were reported as numbers and percentages with 95% confidence intervals. Relationships between variables were explored using Pearson’s correlation (two tail) for normally distributed data (use of CM) and Spearman’s Rho correlations or Gamma co-efficient for non-parametric measurements (type of CM practitioner). Logistic regression was used to predict whether or not perceptions of effectiveness (dependent variables) were associated with reasons for use (covariates). A value of 0.05 was considered statistically significant with 95% confidence intervals.

Non-response bias was assessed using the continuum of resistance model; this involves late responders viewed by proxy as non-responders due to similar characteristics [13]. This method of assessment was chosen due to the higher likelihood for early response by women who found the topic salient or interesting [14]. Responses to the survey four months after the final reminder were classified as late responders and compared for difference with early responses to assess the impact of non-response bias.

## Results

### Response rate

Four hundred and ninety three (493) women with PCOS responded to the survey. A response was received from 235 invited members of the consumer support group POSAA (response rate of 91.1%). An additional 311 women who responded to the survey used the University Research Facebook group.

The majority of participants were aged between 25 and 34 years, born in Australia, attained tertiary qualifications, were employed and held private health insurance (Table 1).

**Table 1:** **Demographic characteristics of respondents**

	N = 493	%	95% CI
Age			
17 or less	3	0.7	± 0.79
18-24	72	17.0	± 3.58
25-29	126	29.7	± 4.35
30-34	111	26.2	± 4.19
35-40	71	16.7	± 3.55
41-44	25	5.9	± 2.24
45+	16	3.8	± 1.82
*Missing data*	69		
Education			
*Completed high School*			
Yes	371	87.5	± 3.15
*Missing data*	69		
*Completed tertiary education*			
Yes	323	76.2	± 4.05
*Missing data*	69		
Qualification from TAFE	103	20.9	± 3.59
Private institution Diploma	70	14.2	± 3.08
University degree	221	44.8	± 4.39
*Missing data*	99		
*Ethnicity- country of birth*			
Australia	302	61.3	± 4.3
Other	121	24.5	± 2.08
*Missing data*	70		
Employment			
Home duties	117	23.7	± 3.75
Self-employed	38	7.7	± 2.35
Student	80	16.2	± 3.25
Employed part time	103	20.9	± 3.59
Employed full-time	194	39.4	± 4.31
Othe*r*	12	2.4	± 1.35
*Missing data*	68		
Private Health Insurance			
Yes	275	65	± 4.55
***Missing data***	**70**		

*Missing data* for age, complete high school, tertiary education, ethnicity, employment and health insurance was 14.0%. *Missing data* for qualifications 20.1%.

Women reported multiple symptoms of PCOS. The most common included late menses (69%, ±4.31, 95% CI); hirsutism (66%, ±4.20, 95% CI); acne (51%,±4.52, 95% CI); insulin imbalances ( 56%, ±4.63, 95% CI), being overweight (78%, ±4.46, 95% CI) or very overweight, (51%, ±4.61, 95% CI). Other health complaints included depression (45%, ±4.64, 95% CI) and infertility (53%, ±4.49, 95% CI).

The respondents used various medical therapies to manage their symptoms. Over 65% (±4.21, 95% CI) had used the oral contraceptive pill; 62.7% (±4.26, 95% CI) had used other pharmaceuticals including ovulation induction, hypoglycaemic and anti-androgen drugs. Medications used to stimulate the ovaries for IVF were reported by 11.8% (±2.87, 95% CI).

### Use of complementary medicines and therapies

Three hundred and four, out of 432 women (70.4% ±4.30 95% CI) reported using complementary medicine (CM) in the previous 12 months.

Women were asked about their intake habits (Table 2). The most commonly used CM products taken on a daily basis were dietary supplements including vitamins, minerals and food nutrients such as fish oil capsules, and herbal supplements. Two hundred and seven of 432 respondents (47.9% ±4.71 95% CI) reported use of more than one type of CM product.

**Table 2:** **Types of ingestible complementary medicine taken by participants and frequency of use**

***Complementary medicine use N = 432***	Daily use		Weekly use		Total use		95% CI
	N	%	N	%	N	%	
Nutritional supplements, including vitamins, minerals, nutrients from food such as fish oil.	192	44.4	92	21.3	246	56.9	± 4.67
Herbal medicines including herbal tea (for therapeutic reasons), tablets and liquids.	138	31.94	55	12.73	181	41.9	± 4.65
Vitamins and/or minerals prescribed by a medical doctor	62	14.4	10	2.3	63	14.6	± 3.33
Multiple forms of CAM products	137	31.71	70	16.2	207	47.9	± 4.71
Total use	238	55.1	179	41.4	304	70.4	± 4.3

● Categories were not mutually exclusive, women may have been using more than one product/modality with variable patterns of use.

● CI confidence interval.

### Complementary practitioner visits

Consultations with acupuncturists, chiropractors, naturopaths, and massage therapists in the previous twelve months were reported by 233 of 304 CM users (76.6% ± 4.76 95% CI) (Table 3) . A smaller number of women also consulted medical doctors for acupuncture treatment or integrative medicine which was provided within the general medical setting. Nearly half of respondents using CM consulted multiple CM practitioners during the previous twelve months, usually an acupuncturist and naturopath (n = 47, 15.5% ±4.07 95% CI) or an acupuncturist and chiropractor (n = 41, 13.5% ±3.84 95% CI).

**Table 3:** **Consultations with a complementary practitioner during the previous twelve months**

***Use of CM***	N	%	95% CI
***N = 304***			
Consultations with CM practitioners	233	76.6	± 4.76
More than one type of CM practitioner	136	44.7	± 5.59
Naturopath	118	38.8	± 5.48
Chiropractor	103	33.9	± 5.32
Acupuncturist	88	28.9	± 5.1
Massage therapist	79	25.1	± 4.87
Osteopath	41	13.5	± 3.84
Reflexologist, aromatherapist, iridologist	36	11.8	± 3.63
Homoeopath	34	11.2	± 3.55
Medical doctor for integrative medicine	27	8.9	± 3.2
Kinesiologist	20	6.6	± 2.79
Medical doctor for acupuncture	14	4.6	± 2.35

• Footnote: 301 women responded to this question, missing data = 3.

• Responses were not limited to single options with 136 providing more than one response for type of CM practitioner.

• CI, confidence interval.

### Reasons for using Complementary Medicine (CM)

Many women were using CM to treat PCOS (n = 199, 65.5%, ± 5.34, 95% CI) however the majority of participants reported using CM to assist with improving more than one aspect of their health (n = 277, 76.7%, ± 4.75, 95% CI). The most common reasons for use were to improve general wellbeing (n = 135, 44.4% ± 5.59 95% CI); and to treat PCOS symptoms and infertility (n = 102, 33.6%, ± 5.31, 95% CI), and treat PCOS and reduce depression (n = 92, 30.3% ± 5.17 95% CI).

### Views about CM

Twenty seven percent of CM users (n = 82, ± 5.99, 95% CI) indicated they thought CM used alone was effective management for PCOS and 24.7% (n = 75, ±4.85, 95% CI) reported that CM was effective for PCOS in conjunction with diet and exercise. Participant’s indicated they thought CM was effective for treating more than one condition, (n = 115, 37.8%, ± 5.44, 95% CI) including improved general well-being (n = 96, 31.6%,± 5.23 95% CI), sleep ( n = 51, 16.8%,±4.22 95% CI) and weight loss (n = 48, 15.8%,± 4.09 95% CI). A smaller number of women considered CM was effective for infertility (n = 33, 10.9% ± 3.5 95% CI).

Significant relationships were found between using CM to treat specific conditions and perceptions of effectiveness. Use of CM to treat PCOS (n = 199), was associated with effectiveness of CM by 40.8% (PCC 4.64, p = 0.001) of participants. Use of CM as treatment for infertility (n = 102) was associated with effectiveness by 32.4% (PCC 3.84, p = 0.001) of women and the treatment goal, ‘to improve wellbeing’ (n = 134), was associated with 71.6% (PCC 4.04, p = 0.001) of women reporting effectiveness.

Predictions were found for using CM to treat PCOS and perceptions of effectiveness for PCOS (odds ratio (OR) 24.8, CI 11.1-55.3, p = 0.0001); infertility, (OR 18.8, CI 7.9-44.8, p = 0.0001) and for improved wellbeing (OR 8.8, CI 5.2-14.9, p = 0.0001).

Advantages for CM were reported by 52.5% (n = 259, ± 4.41, 95% CI). These included perceived naturalness of CM (n = 157, 60.6% ± 5.95, 95% CI), the complementary nature of treatment (n = 120, 46.3% ± 6.07, 95% CI), perceived low risk of side effects (n = 104, 40.2%, ± 5.97, 95% CI), increased self- responsibility (n = 81, 31.3%, ± 5.65, 95% CI) and the holistic nature of CM (n = 75, 29%, ± 5.52 95% CI).

Disadvantages associated with CM use were cited by 354 women (71.8%, ± 3.97 95% CI). These included CM being too expensive (n = 187, 52.8% ± 5.2, 95% CI), lack of research into CM (n = 175, 49.4% ± 5.21 95% CI) and 163 women (46.0% ± 5.19, 95%CI) were not confident about a benefit from CM. A small number of women (13.6%, ± 3.57, 95% CI) indicated they were not confident to use CM in conjunction with other pharmaceuticals.

### Adverse reactions and CM

Participants were asked about any adverse reactions or negative experiences with their use of CM. Adverse reactions to nutritional and herbal supplements, Traditional Chinese Herbal Medicine (TCHM) and acupuncture were reported by thirty seven CM users (12.2%, ±3.68, 95% CI). Nine reactions were to vitamins, seven to fish oil supplements, six to TCM including two to acupuncture, two to herbal teas and five to vitamin supplements prescribed by a medical practitioner. Eleven adverse reactions were reported including longer menstrual cycles, changes to bowel habits, sleep disturbances and headaches.

### Assessment for non-response bias and selection bias

When testing for non-response bias for early and late responders no significant differences were noted in the demographic characteristics, symptoms of PCOS and prevalence for use of CM between any of the surveyed groups.

## Discussion

The use of complementary medicine by women has increased in recent years. We present the first survey of women with PCOS and report high use of CM, with nearly three out of four women reporting use and nearly half reporting use of multiple CM’s. This study demonstrates that women seek more than the management of symptoms associated with PCOS when using CM’s and that well-being, reproductive and mental health are important aspects of care. Our findings are limited by the sampling framework and the referral of participants through support groups, since women with PCOS whom have not sought support through POSAA or electronic social media, or women with PCOS in the community who have not been diagnosed [1] were not represented in this study.

We found women have similar preferences for the types of CM use to those reported in reproductive health settings where nutritional and herbal supplements were the most commonly reported CM (comparatively, 56% for women attending family planning clinics and 78% of women attending fertility clinics) [7, 15]. However, our findings differ to studies showing recently increased preferences for acupuncture compared to naturopaths [7, 16]. These differences may be explained by our study focusing only on women with PCOS, the definition of CM modalities used or influenced by recent statutory registration of acupuncture practitioners in Australia.

Most women perceived the effectiveness of CM as at least somewhat helpful. This aligns with findings from other surveys of women attending family planning clinics [7], pregnant women [17] and middle aged women [8]. Our data show women’s perceptions of effectiveness were increased when they used CM with specific treatment goals in mind. Little is known about how women with PCOS acquire information and make decisions regarding suitable CM’s. This extended beyond the scope of our study however our findings demonstrate the value of informed CM treatment decisions and highlight the decision process for CM as an area of future research need. The main disadvantage for CM cited in this study was the lack of research which reiterates women’s need for unbiased information to support their CM decisions.

Our data corresponds with findings reporting women have reduced recognition for potential hazards for CM [7, 18, 19]. Some herbal medicines taken in conjunction with pharmaceuticals may have adverse effects. *Hypericum perforatum* (St Johns wort) for example, has been shown to reduce pharmaceutical effectiveness [20–22]. Importantly, our findings report participant’s high use for herbal medicines and high use of pharmaceuticals including the oral contraceptive pill (over 65%). Future research is needed to examine concurrent use of pharmaceuticals and CM by women with PCOS, and the rate of adverse events attributable to reduced pharmaceutical effectiveness.

The rate of adverse events reported here was not as low as in other studies. A survey of TCM (Chinese herbal medicine and acupuncture) practitioners demonstrated adverse reaction rates of one per 633 consultations [23]. Comparatively there were six adverse reactions for TCM out of 304 CM users. Our data include self-reported adverse reactions by self-prescribing consumers with potentially poorly informed treatment choices. This may explain the higher rates of adverse reactions in this case. On the other hand, adverse reactions reported by TCM practitioners may be at risk of reporting bias. Our research highlights that women with PCOS do experience adverse reactions when using CM, despite perceptions of low risk.

This study does have some methodological limitations. We were unable to directly assess non-response and selection bias for the Facebook group. The referral to the survey by administrators of other Facebook support groups limited our ability to gauge unsolicited invitations and data were not available for assessment using the continuum of resistance model. Generalizations are limited to computer literate women with English language skills and other groups of women may be under-represented. However the results may be generalisable to women with PCOS. Women reported similar prevalence for key PCOS signs and symptoms to clinical populations for hirsutism (55.6% [1], 66% [24] and 75% [25]) and menstrual irregularities (62.5% [1], 66% [24] and 89% [25]). This suggests that the respondents resemble a clinical population with PCOS. We also attained a good response rate without response bias from women with PCOS in a non-clinical setting, not restricted by geographical location. This survey had high completion rates and suggests that the methods were acceptable to women. For these reasons our data are likely to reflect the authentic views of women with PCOS in the community. This research was focused on the use of CM by women with PCOS and differentiates CM from other lifestyle practices; however women often use CM within the context of other lifestyle behaviors, including diet and exercise. The prevalence, patterns and attitudes towards diet and exercise of women with PCOS was beyond the scope of the present article.

## Conclusion

Complementary medicine may present a treatment option with high acceptance by women; this study is an important step in developing our understanding of this group of women’s views and patterns of CM use.

## Electronic supplementary material

Additional file 1: **The survey instrument.** (PDF 223 KB)
