# Supplementary material for: A survey of the use of complementary medicine by a self-selected community group of Australian women with polycystic ovary syndrome
Source: BMC Complement Altern Med. 2014 Dec 6;14:472. doi: 10.1186/1472-6882-14-472 (PMC4265410; doi:10.1186/1472-6882-14-472)
Supplement: Supplementary file 1 — Additional file 1: The survey instrument. (PDF 223 KB) [file 12906_2014_2035_MOESM1_ESM.pdf]

Polycystic ovary syndrome (PCOS), self help and complementary

Polycystic ovary syndrome (PCOS), self help and complementary medicine; the...

This survey is part of a study is being carried out by the University of Western Sydney with the support of the Polycystic Ovary Association of Australia (POSAA) to provide a clearer understanding of the use of self prescribed therapies and complementary medicine or therapies by women with polycystic ovary syndrome (PCOS). Your answers will help to better understand the experiences of women with PCOS and for health providers to provide better care. The main therapies in this questionnaire include diet, exercise and physical activity and complementary medicine and therapies. Many complementary medicines do not have a clear evidence base for their effectiveness and this study does not in any way imply that POSAA endorse or refute the use of these therapies.
Your help would be appreciated in the completion of this anonymous survey. Your involvement is voluntary and returning the completed questionnaire will indicate your consent to participate in this study. It should take about 20 minutes to complete the questionnaire. Please note that after submission, your survey cannot be withdrawn. The survey is confidential and the answers given by you are not identifiable.
When completing the survey please click on the box which best represents your answer. When you finish the survey, please click on the submit button. There are no right or wrong answers. The results will be published on the Polycystic Ovary Syndrome Association of Australia (POSAA) website.
This study is being carried out by a PhD student at the Centre for Complementary Medicine Research at the University of Western Sydney. It has been approved by the Human Research Ethics Committee of the University of Western Sydney, approval number H9341.
If you have any questions regarding the survey please contact the researcher, Susan Arentz on 0403 044 247 or email s.arentz@uws.edu.au. If you have any concerns or complaints about the ethical conduct or content of the survey, please contact the Office of Research Services at humanethics@uws.edu.au
Thank you for your participation.

What is Complementary Medicine?
Complementary alternative or natural medicine or therapies includes herbal tablets or capsules, vitamins and/or mineral supplements, food supplements such as fish oil or barley greens or visiting a complementary, traditional medicine or alternative practitioner. In this survey, complementary medicine or therapies is referred to as CM.

To start we would like to ask you some questions about your general health and the way you expeience PCOS.

1. In general, would you say that your physical health is (choose one only)

- ◯ Excellent
- ◯ Very good
- ◯ Good
- ◯ Fair
- ◯ Poor

2. Do you experience any signs or symptoms that may be associated with PCOS?

- ◯ Yes
- ◯ No (please go to question 4)

3. Do you experience any of the following signs or symptoms that may be associated with PCOS (choose as many as needed)

	All of the time	Most of the time	Some of the time	A little of the time	Hardly any of the time	Not any of the time
A late menstrual period	◯	◯	◯	◯	◯	◯
Menstrual cramps	◯	◯	◯	◯	◯	◯
Skin breakouts or skin irritation on the face, back or chest	◯	◯	◯	◯	◯	◯
Depressed as a result of having PCOS	◯	◯	◯	◯	◯	◯
Growth of visible hair on chin or body	◯	◯	◯	◯	◯	◯
Waist larger than hips	◯	◯	◯	◯	◯	◯
Overweight	◯	◯	◯	◯	◯	◯
Very overweight	◯	◯	◯	◯	◯	◯
Metabolic syndrome or insulin or sugar imbalances	◯	◯	◯	◯	◯	◯
Infertility, or taking longer than 12 months to get pregnant	◯	◯	◯	◯	◯	◯

Other (please specify)

4. Do you keep a record of your menstrual cycle?

◯ Yes - always

◯ Yes - sometimes

◯ No

5. Have you tried any of the following to reduce or manage PCOS symptoms (please tick as many as needed)

☐ Exercise/physical activity

☐ Dietary changes

☐ Complementary medicine or natural therapies (CM)

☐ Pharmaceuticals or drugs such as Metformin or Clomid (Clomiphene citrate)

☐ The oral contraceptive pill

☐ Fertility drugs as part of an IVF cycle

☐ Surgery for PCOS such as laparoscopic ovarian drilling or bariatric (stomach) surgery

☐ No I've not tried any of these things to reduce or manage PCOS

Other (please specify)

Self help and complementary medicine or natural therapies for PCOS

Now we want to ask you in more detail about ways, if any, that you have tried to keep yourself well or to manage the symptoms of PCOS. First some questions about complementary medicine or natural therapies, followed by some questions about diet and lastly some questions about exercise or physical activity.

6. Do you, or have you ever taken complementary medicines or natural therapies such as vitamins, minerals, herbs etc?

- ○ Yes
- ○ No (please go to question 8)

7. In the past 12 months or do you currently use or take any of the following complementary medicines or natural therapies (tick as many as needed)

	How often?
Vitamins such as multivitamins, or vitamin C, not prescribed by a doctor	
Other natural medicines from fish, plants or animals	
Herbal teas, for therapeutic reasons	
Mineral supplements not prescribed by a doctor	
Food supplements such as spirilina, barley greens etc	
Mineral supplements prescribed by a doctor	
Herbal medicines such as Vitex agnes castus (chaste tree), dong quai, Echinacea etc	
Other herbal medicines	
Traditional Chinese herbal tablets or teas	
Indigenous or other traditional medicines such as Ayevedic medicine	
Vitamins prescribed by a doctor	
Aromatherapy oils for therapeutic reasons	
Homoeopathic medicines	
No complementary medicines or natural health supplements used in the last 12 months	

Other (please specify)

8. Have you ever visited or are you currently seeing any of the following complementary practitioners or therapists (please tick as many as needed)

- ☐ Acupuncturist
- ☐ Chiropractor
- ☐ Naturopath/western herbalist
- ☐ Osteopath
- ☐ Homoeopath
- ☐ Traditional Chinese Medicine practitioner
- ☐ Reflexologist
- ☐ Massage or Bowen therapist
- ☐ Aromatherapist
- ☐ Iridologist
- ☐ Kinesiologist
- ☐ A medical doctor for acupuncture
- ☐ A medical doctor for integrative medicine
- ☐ No I have not visited a complementary practitioner or therapist. If you have not used, are not currently using and have not visited a complementary practitioner or therapist please go to question 13.

Other (please specify)

9. For what area of your health have you used, currently use complementary medicines or therapies or see a complementary therapist or practitioner (please tick as many as needed)

- [ ] PCOS
- [ ] Increase energy
- [ ] Period pain
- [ ] PMS
- [ ] Fertility
- [ ] Sleep
- [ ] Digestion
- [ ] Constipation
- [ ] Acne
- [ ] Colds and flu prevention
- [ ] Stay healthy
- [ ] Depression
- [ ] Anxiety
- [ ] General well-being

Other (please specify)

10. Have you found complementary medicines or therapies effective for (please tick as many as required)

- [ ] Reducing PCOS symptoms such as regulating menstruation or reducing acne
- [ ] Improving PCOS in combination with diet and exercise
- [ ] Improving PCOS in combination with drugs
- [ ] Reducing period pain
- [ ] Reducing PMS
- [ ] Improving general health
- [ ] Improving fertility
- [ ] Improving sleep
- [ ] Enhancing weight loss
- [ ] No not effective at all

Other (please specify)

11. If you are using CM's for PCOS, what or who most influenced your decision to use complementary therapies (please tick as many as needed)

- [ ] I self prescribed
- [ ] Internet
- [ ] Retail assistant
- [ ] Family or friends
- [ ] Medical doctor
- [ ] Complementary medicine practitioner such as a naturopath, herbalist or acupuncturist
- [ ] Health professional such as a nurse, chemist, psychologist, physiotherapist or other health provider
- [ ] Vitamin company hotline
- [ ] Magazines or books
- [ ] TV

Other (please specify)

____

12. What do you think are the advantages of CM's (please tick as many as needed)

- [ ] It's more natural
- [ ] Lack of side effects
- [ ] Safe
- [ ] Effective, it seems to work
- [ ] Lasting effects
- [ ] Holistic effects
- [ ] Not addictive
- [ ] Can complement existing health care
- [ ] Can't do any harm
- [ ] I can be responsible for my own health and well-being
- [ ] I have not tried, so not sure

Other (please specify)

____

13. What do you think are the main disadvantages of using CM's (please tick as many as needed)

- [ ] I don't think there are any
- [ ] Expensive
- [ ] Takes longer to work
- [ ] Lack of research on whether or not it works
- [ ] Lack of research on safety such as no warnings
- [ ] Not sure if it will work
- [ ] Not enough information
- [ ] Taste or smell of preparations
- [ ] Not confident to use in conjunction with other drugs
- [ ] Not prescribed or recommended by my doctor
- [ ] Don't know

Other (please specify)

14. Have you ever had a negative reaction or unwanted side-effects to any complementary medicine or therapies?

- ( ) Yes
- ( ) No (please go to question 17)

15. If you have experienced negative or unwanted side effects, to which complementary medicines or therapies (please tick as many as needed)

- ☐ Vitamins such as multivitamins or vitamin C, not prescribed by a doctor
- ☐ Other natural medicines from fish, plants or animals
- ☐ Herbal teas for therapeutic reasons
- ☐ Mineral supplements not prescribed by a doctor
- ☐ Food supplements such as spirilina, barley greens etc
- ☐ Mineral supplements prescribed by a doctor
- ☐ Herbal medicines such as Vitex agnes castus (chaste tree), dong quai, Echinacea etc
- ☐ Other herbal medicines
- ☐ Traditional Chinese herbal tablets or teas
- ☐ Indigenous or other traditional medicines such as Ayevedic medicine
- ☐ Vitamins prescribed by a doctor
- ☐ Acupuncture
- ☐ Reflexology
- ☐ Kinesiology
- ☐ Iridology
- ☐ Massage or Bowen therapy
- ☐ Aromatherapy oils for therapeutic reasons
- ☐ Homoeopathy or homoeopathic medicines

Other (please specify)

16. If yes, what was the nature of the negative or unwanted side-effects you experienced (please tick as many as needed)

	Temporary and I continued to use	Temporary, side-effects went after I stopped taking the CM	Unwanted side-effects continued after I stopped taking the CM
Menstrual cycle lengthened	◯	◯	◯
Menstrual cycle changed in a way I didn't want	◯	◯	◯
Changes in bowel habits either looser or constipated	◯	◯	◯
Headaches	◯	◯	◯
Vomitting	◯	◯	◯
Skin rashes	◯	◯	◯
Sleep changes	◯	◯	◯
Heart beating fast	◯	◯	◯
Excessive sweating	◯	◯	◯
An anaphylactic reaction	◯	◯	◯

Other (please specify)

Diet or food choices

Next we want to know if you have tried any diets to improve the symptoms of PCOS or to help with weight loss or general well-being.

17. In the past 5 years have you used any diets to improve PCOS?

- ◯ Yes
- ◯ I eat a special diet but not specifically to manage or improve PCOS
- ◯ I eat a healthy diet but not for any particular reason (please go to question 20)
- ◯ No, I'm not interested in diets (please go to question 20)

18. What was the purpose or reason for using the diet (tick as many as needed)

- ☐ To lose weight
- ☐ To improve body composition (eg build muscle mass)
- ☐ To improve health
- ☐ To address a health concern (such as high blood sugar, food allergy or high cholesterol)
- ☐ For social reasons, that is to enjoy with family or friends for example to encourage a family member to eat a particular diet

Other (please specify)

19. Have you tried any of the following diets (tick as many as needed)

- [ ] Low glycaemic (low GI) as in foods that lower blood sugar or glucose or lower insulin
- [ ] Low calorie
- [ ] Low fat
- [ ] Energy restricting diet
- [ ] A whole food diet
- [ ] Healthy eating plan such as the government endorsed lifescripts
- [ ] High protein
- [ ] Low carbohydrate
- [ ] CSIRO
- [ ] Weight Watchers
- [ ] Liver detox diet
- [ ] Palm Beach diet
- [ ] Blood group diet
- [ ] Anti-candida diet
- [ ] Lean cuisine
- [ ] Lite and Easy
- [ ] Protein powders
- [ ] Meal replacement
- [ ] Very low calorie diet
- [ ] Low allergy diet (gluten or dairy free for example)

Other (please specify)

20. If you were or currently eat a special diet, for a particular reason, do you feel you achieve or achieved your goals?

- ◯ Yes
- ◯ Partly
- ◯ No
- ◯ Don't know

21. Have you experienced weight loss or weight management (prevention of weight gain) as a result of making dietary changes?

- [ ] Yes
- [ ] No but I have experienced positive changes other than weight loss
- [ ] No, no weight loss, no prevention of weight gain and no other positive changes

Other (please specify)

Exercise or physical activity

Next are some questions about exercise and physical activity, both moderate and vigorous activity. Moderate activity is movement that causes a slight increase in your breathing and heart rate, but you should still be able to talk. A brisk walk, mowing the lawn or vacuuming are examples of moderate activity.

22. Do you participate in moderate or vigorous exercise or physical activity?

- ◯ Yes
- ◯ No (please go to question 30)

23. How many days per week do you participate in exercise or physical activity

	more than once per day	every day	5-6 times per week	2-4 times per week	once per week	once or twice per month	I don't do this type of exercise
moderate activity (such as walking)	◯	◯	◯	◯	◯	◯	◯
vigorous activity (such as aerobics, circuit training and running)	◯	◯	◯	◯	◯	◯	◯

24. Regarding moderate activity (such as walking) in a typical day how many hours would you undertake moderate activity

- ◯ I don't do moderate physical activity
- ◯ About a quarter of an hour (15 minutes)
- ◯ About half an hour (30 minutes)
- ◯ About one hour (60 minutes)
- ◯ More than an hour

Other (please specify)

[ ]

25. Regarding vigorous activity (such as aerobics, circuit training and running), for how many hours is your exercise session

- ◯ I don't do vigorous physical activity
- ◯ About a quarter of an hour (15 minutes)
- ◯ About half and hour (30 minutes)
- ◯ About one hour (60 minutes)
- ◯ More than one hour

Other (please specify)

[ ]

26. In which forms of physical activity do you currently participate (tick as many as needed)

- [ ] Lift weights
- [ ] Brisk walking up hills or stairs (where your breathing is heavy, panting and puffing)
- [ ] Run
- [ ] Group fitness classes at gym eg. spinn, zumba, pump
- [ ] Swim
- [ ] Yoga
- [ ] Pilates
- [ ] Personal trainer
- [ ] Dance
- [ ] Cycle
- [ ] Team sports such as netball, hockey etc
- [ ] Tennis (including table tennis)
- [ ] Incidental exercise (as part of a busy lifestyle for example walking up stairs instead of taking the lift, parking the car a long way from your destination, gardening etc)

Other (please specify)

27. Why do you undertake these forms of physical activity (please tick as many as needed)

- [ ] To feel better in myself
- [ ] To develop strength
- [ ] To lose weight
- [ ] To prevent weight gain
- [ ] I was advised to exercise by a health professional
- [ ] Family and friends persuaded me to exercise
- [ ] To meet specific goals or targets
- [ ] To get out of the house
- [ ] To socialise
- [ ] To set an example for my kids, family and/or friends
- [ ] To manage PCOS

Other (please specify)

28. Do you feel that you have achieved your health goals with your exercise and physical activity (please tick one)

- ◯ Yes
- ◯ Partly
- ◯ No
- ◯ Don't know

Other (please specify)

[ ]

29. If physical activity is an ongoing part of your lifestyle, do you expect to be exercising this time next year (please choose one)

- ◯ yes
- ◯ no
- ◯ don't know

30. If you do not participate in moderate or vigorous activity, is there a particular reason (please tick as many as needed)

- ☐ Limited time
- ☐ Not done before
- ☐ Physical injury
- ☐ Feel self conscious or embarrassed
- ☐ No difference to my health
- ☐ No difference to my weight
- ☐ Increased my weight
- ☐ Trying to get pregnant
- ☐ I am pregnant
- ☐ Not convinced of the benefits
- ☐ Limited money

Other (please specify)

[ ]

Describe yourself

Finally some questions about you, to help us describe the women who have taken part in the survey.

31. Are you aged between

- ○ 17 or less than 17
- ○ 18 - 19
- ○ 20-24
- ○ 25-29
- ○ 30-34
- ○ 35-40
- ○ 40-44
- ○ 45+

32. Did you finish high school

- ○ yes
- ○ no
- ○ still completing high school education

33. Have you completed any tertiary education

- ○ yes
- ○ no
- ○ Currently completing tertiary education

34. If yes indicate if the qualification was (tick as many as needed)

- ☐ A qualification from TAFE or similar
- ☐ Diploma
- ☐ University degree

35. In which country were you born?

- ○ Australia

Other (please specify)

____

36. Which of the following best describes your working activities (tick as many as required)

- [ ] Home duties
- [ ] Self employed
- [ ] Student
- [ ] Employed part-time
- [ ] Employed full-time

Other (please specify)

37. Do you have private health insurance?

- ( ) yes
- ( ) no

Thank you for your help. Please remember to click on the submit button at the bottom of this page.
